# MOFs Derived Hetero-ZnO/Fe_2_O_3_ Nanoflowers with Enhanced Photocatalytic Performance towards Efficient Degradation of Organic Dyes

**DOI:** 10.3390/nano11123239

**Published:** 2021-11-29

**Authors:** Fakhr uz Zaman, Bing Xie, Jinyang Zhang, Tianyu Gong, Kai Cui, Linrui Hou, Jiali Xu, Zhirou Zhai, Changzhou Yuan

**Affiliations:** School of Materials Science & Engineering, University of Jinan, Jinan 250022, China; Azaman_ME@163.com (F.u.Z.); Axieb_ME@163.com (B.X.); Azhangjy_ME@163.com (J.Z.); Agongty_ME@163.com (T.G.); Acuik_ME@163.com (K.C.); Axujl_ME@163.com (J.X.); Azhaizr_ME@163.com (Z.Z.)

**Keywords:** ZnO/Fe_2_O_3_ nanoflowers, junction structures, metal-organic frameworks, photocatalytic degradation, organic dyes

## Abstract

It is still a challenge for wastewater treatment to develop efficient yet low-cost photocatalysts on a large scale. Herein, a facile yet efficient method was devised to successfully synthesize ZnO/Fe_2_O_3_ nanoflowers (NFs) by using metal organic framework ZIF-8 as the precursor. The photocatalytic activities of the as-prepared hetero-ZnO/Fe_2_O_3_ NFs are purposefully evaluated by photocatalytic degradation of methylene blue (MB) and methyl orange (MO) under UV light irradiation. The resulting ZnO/Fe_2_O_3_ NFs display even higher photocatalytic activities than those of single-phase ZnO and Fe_2_O_3_ as a photocatalyst for the degradation of both MB ad MO. Particularly, nearly 100% MB can be photocatalytically degraded in 90 min under UV light irradiation using the hetero-NFs photocatalyst. The enhanced photocatalytic properties are probably ascribed to the synergistic contributions from the suitable band alignment of ZnO and Fe_2_O_3_, large surface area, and strong light absorption property. Radical scavenger experiments prove that the photogenerated holes, ·OH and ·O_2_-, play key roles in photocatalytic degradation process of organic dyes. Accordingly, the photocatalytic degradation mechanism of hetero-ZnO/Fe_2_O_3_ NFs towards dyes is tentatively proposed. The work contributes an effective way to rationally design and fabricate advanced photocatalysts with heterojunction structures for photocatalytic applications.

## 1. Introduction

In recent years, with the rapid increase in population and fast development of industries, the environmental issues have become more serious. In particular, organic dyes in textile and industrial effluents would cause serious pollution to the environment if they were directly poured into the water body. However, some traditional treatment technologies, such as adsorption, flocculation, and biological degradation, do not work efficiently [1,2]. Therefore, the efficient treatment of dye wastewater has become an indispensable yet challenging topic in our daily life.

Recently, photocatalysis using semiconductors has emerged as an effective method for wastewater treatment and environmental remediation [3,4]. Among all semiconductor photocatalysts employed in water purification, the semiconductor of ZnO has received a lot of attention due to its direct band gap, non-toxicity, high photocatalytic activities, good carrier mobility, and lower cost [5,6,7]. Despite these inherent merits of ZnO, the rapid recombination of photogenerated carriers within the semiconductor and larger band gap may result in low efficiency, thus hindering its potential applications [8,9,10]. In view of this, typical composites with heterostructures have been developed as an efficient platform to address the recombination of photogenerated electrons and holes, as well as the utilization rate of UV/visible light in ZnO-based photocatalysts [11,12,13,14]. The incorporation of small band gap semiconductors, cocatalysts, and dyes is normally adopted to extend the absorption wavelength range of a semiconductor from the UV light to the visible light region [15,16,17]. In comparison to the single-phase ZnO, the coupled semiconductor systems demonstrate even higher photocatalytic activities.

Besides the synergetic effects of different components, the suitable morphology/structure characters of composites can effectively improve their photocatalytic performance as well. To ensure as many contactable sites of photocatalysts with target pollutants as possible, porous materials with well-distributed metal-based active sites are particularly preferred. Metal-organic frameworks (MOFs), assembled by metal ions and organic ligands through coordination bonds, are a new class of highly crystalline porous materials with unique advantages, such as tunable porosity, high surface area, and so on [18,19,20]. Especially in recent years, MOFs have been widely applied as promising templates and/or precursors to prepare metal oxide-based porous materials for applications in photocatalysis, sensors, and energy storage fields [21,22,23]. Among numerous MOFs reported before, the zeolitic imidazolate frameworks (ZIFs) are widely studied as the isostructural porous materials, and ZIF-8 is one of the most representative MOFs due to its robust chemical and thermal stabilities [19,20].

In this work, we devised a MOFs-derived method to successfully prepare the hetero ZnO/Fe_2_O_3_ nanoflowers (NFs) by using the ZIF-8 as a precursor. The purpose of the design here is to combine the advantages of both hierarchical porous structure and heterojunction. The photocatalytic performance of the as-prepared ZnO/Fe_2_O_3_ NFs was evaluated in detail under UV light irradiation by choosing organic dyes of methylene blue (MB) and methyl orange (MO) as model pollutants, respectively. The resultant ZnO/Fe_2_O_3_ NFs demonstrated appealing photocatalytic activities and stability towards the photocatalytic degradation of both MB and MO. Furthermore, the underlying mechanism of enhanced photocatalytic activities for the ZnO/Fe_2_O_3_ composite was also tentatively put forward based on the calculated energy positions of the ZnO and Fe_2_O_3_.

## 2. Experimental Section

### 2.1. Materials

Zinc acetate dehydrate (Zn(AC)_2_·2H_2_O), ferrous sulfate heptahydrate (FeSO_4_·7H_2_O), 2-methylimidazole (MIM), MB, MO and ethanol (95%) were purchased from Sinopharm Chemical Reagent, Co.,Ltd.(Shanghai, China) and utilized as received without any further purification. The de-ionized (DI) water was used as the solvent.

### 2.2. Synthesis of ZIF-8 and ZnO

ZIF-8 was prepared with a modified procedure reported by Lou and his co-workers [24]. Typically, 0.300 g of Zn(Ac)_2_·2H_2_O was dissolved in 5 mL of DI water to form a clear solution I, and 1.120 g of 2-methylimidazole was dissolved in 5 mL of DI water to obtain solution II. Afterwards, solution I was added into solution II drop by drop under vigorous magnetic stirring. The resulting suspension was kept for 1 h and then placed at room temperature (RT) for another 48 h. Subsequently, the solid products were collected by centrifugation, washed separately with DI water and ethanol several times, and further dried in an oven at 60 °C for 6 h. The phase-pure ZnO was finally prepared by annealing the ZIF-8 in a muffle furnace at 420 °C for 6 h at a ramping rate of 2 °C min^−1^.

### 2.3. Synthesis of ZnO/Fe_2_O_3_ NFs

To synthesize the products, 0.020 g of resulted ZnO was added into 40 mL of DI water and stirred for 30 min for good dispersion, and then it was kept in an ice/H_2_O bath (0 °C). Simultaneously, 0.100 g of FeSO_4_·7H_2_O was added into 10 mL of DI water to obtain a clear solution. Subsequently, the obtained FeSO_4_ solution was added drop by drop under stirring into the above solution with well-dispersed ZnO in the ice/H_2_O bath (0 °C). After 20 min, the precipitate was collected, centrifuged, and washed separately with DI water and ethanol several times. The product was dried in an oven at 80 °C for 12 h, and then the powder was transferred to a muffle furnace and then calcinated at 500 °C for 5 h at the ramping rate of 5 °C min^−1^. For comparison, pure Fe_2_O_3_ was obtained with a similar method as ZnO just with the exception of FeSO_4_·7H_2_O instead of Zn(AC)_2_·2H_2_O and annealed at 700 °C for 5 h at a ramping rate of 5 °C min^−1^.

### 2.4. Material Characterization

The structure and morphology of the samples were investigated with a field emission scanning electron microscope (FESEM, FEI QUANTA FEG250, Munich, Germany), transmission electron microscopy (TEM), scanning TEM (STEM), high-resolution TEM (HRTEM), and selected area electronic diffraction (SAED) (JEOL, System JEM-2100). Additionally, energy dispersive X-ray (EDX) analysis was performed on JEOL JEM-2100F with an X-ray spectrometer attached to the TEM instrument (Japan). The phase composition and crystal structure of products were characterized on a powder X-ray diffraction (XRD) instrument (Rigaku Ultima IV X, Cu K*α* Japan). The Brunauer–Emmett–Teller (BET) specific surface area (SSA) and pore size distribution (PSD) of samples were performed by N_2_ sorption and Barrett–Joyner–Halenda (BJH) methods on Autosorb-IQ/MP surface area analyzer (Quantachrome, America). UV-vis absorption spectra were recorded by using a SHIMADZU UV-2600 spectrophotometer. UV-vis diffused reflectance spectra (DRS) of the samples were taken using a UV-vis spectrophotometer (Shimadzu UV2600, China), and BaSO_4_ was used as a reference reflectance in the UV-vis spectrophotometer, China). The steady-state photoluminescence (PL, Dulshburg F.R.Germany) spectra were reordered by an RF-6000 spectrofluorophotometer. X-ray photoelectron spectroscope (XPS USA) measurements were performed on Thermo ESCALAB 250Xi X-ray photoelectron spectrometer.

### 2.5. Photocatalytic Activity Evaluation

The photocatalytic properties of the as-prepared samples were evaluated by photocatalytic degradation of aqueous MB and MO solutions, respectively. The reactor consisted of a quartz glass beaker (100 mL) and a magnetic stirring setup. Two UV lamps (30 W) were positioned parallelly the sides of the beaker, and their distance to the beaker was 15 cm. The whole photocatalytic reactor was insulated in a stainless-steel box to prevent the escape of harmful radiation and minimize temperature changes caused by draughts. A total of 0.050 g of the resulted photocatalyst was added into 100 mL of a dye solution with an initial mass concentration of 1 mg L^−1^ in the quartz reactor. The solution was stirred for 30 min in the dark to allow the system to reach an adsorption/desorption equilibrium, and then analytical samples were drawn from the reaction suspensions at a given interval during the whole irradiation. The concentrations of the dye solutions were analyzed by a UV-vis spectrophotometer (UV-2600, Japan, and absorption at λ_max_ = 664 nm for MB, and λ_max_ = 464 nm for MO).

## 3. Result and Discussion

### 3.1. Structural and Morphological Characterizations

**Figure 1:**
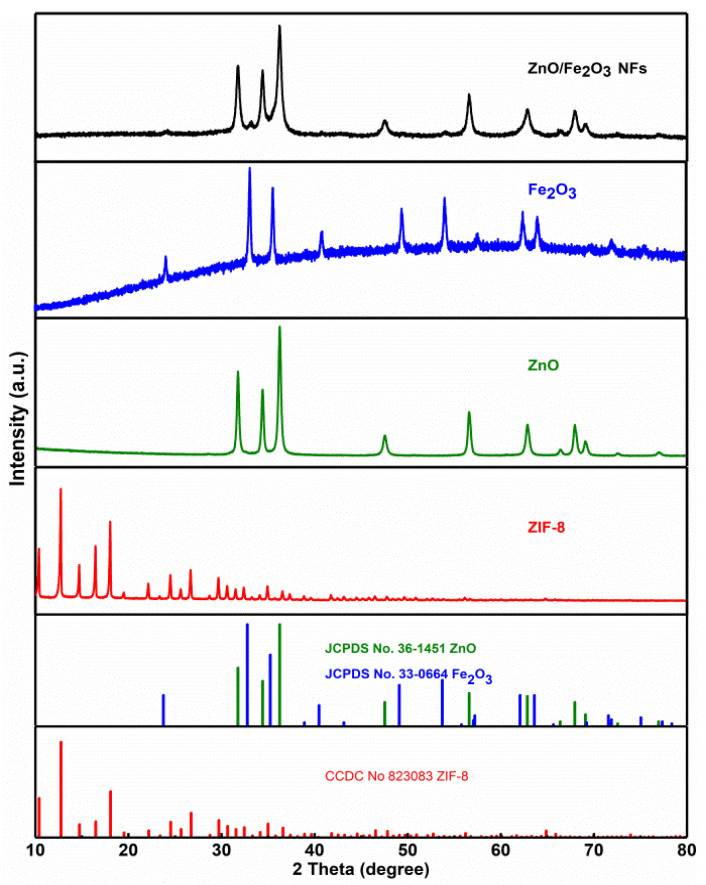
XRD patterns of the ZIF-8, ZnO, Fe_2_O_3_ and ZnO/Fe_2_O_3_ NFs as indicated.

The powder XRD analysis was carried out to identify all samples. Figure 1 comparatively presents the XRD patterns of the ZIF-8, ZnO, Fe_2_O_3_, and ZnO/Fe_2_O_3_ NFs. The diffraction peaks of ZIF-8 are consistent with the standard card (JCPDS No. 823083) [20]. As for the ZnO and Fe_2_O_3_ samples, all the diffraction peaks can be well indexed to the hexagonal ZnO phase (JCPDS No. 36-1451) with lattice parameters (*a* = *b* = 3.25 Å, *c* = 5.21 Å) and the rhombohedral corundum Fe_2_O_3_ phase (JCPDS No. 33-0664) with the lattice parameters (*a* = *b* = 5.03 Å, *c* = 13.75 Å). No characteristic peaks are observed for other impurities. Besides the diffraction peaks of ZnO, other reflections for Fe_2_O_3_ are also detected evidently in ZnO/Fe_2_O_3_ NFs. Compared to pure ZnO, the peak intensity of the ZnO phase in ZnO/Fe_2_O_3_ NFs decreases slightly. Owing to the low crystallinity degree and/or nano-dimension of the Fe_2_O_3_ in the ZnO/Fe_2_O_3_ NFs, the representative peaks originating from the Fe_2_O_3_ turn out to be somewhat weak.

**Figure 2:**
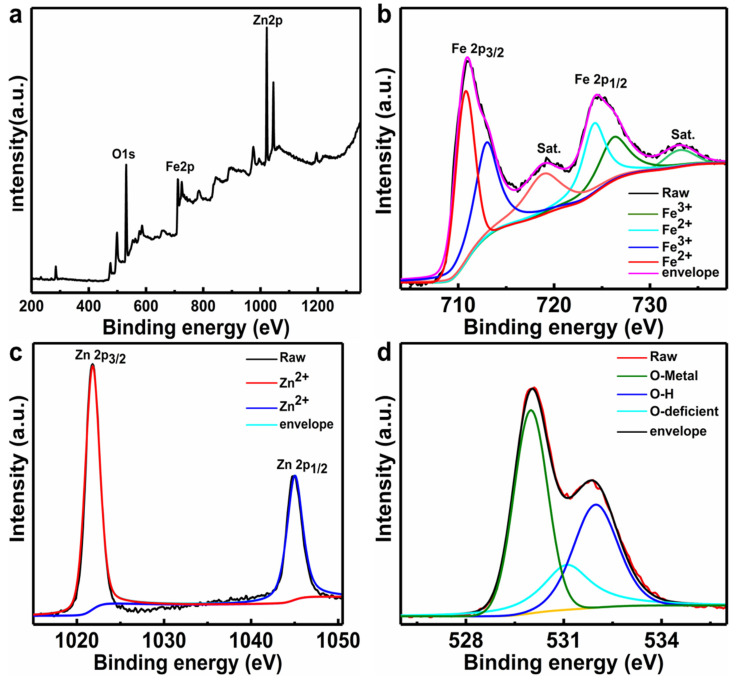
XPS spectra of the ZnO/Fe_2_O_3_ NFs: (**a**) survey spectrum, (**b**) Fe 2p, (**c**) Zn 2p, and (**d**) O 1s.

To further examine the element composition and oxidation states of the as-prepared ZnO/Fe_2_O_3_ NFs, XPS analysis was carried out. The corresponding XPS results are depicted in Figure 2. The characteristic survey spectrum (Figure 2a) indicates the presence of Zn, Fe, and O elements without any other impurities. Based on the Fe 2p, Zn 2p, and O 1s high-resolution spectra, more detailed clarifications for the elemental oxidation states were also conducted. Figure 2b displays the high-resolution spectrum of Fe 2p. The two peaks at binding energies (BEs) of 725.3 and 711.4 eV are assigned to Fe 2p_3/2_ and Fe 2p_1/2_ for the Fe^3+^ [25]. The Zn 2p spectrum (Figure 2c) shows two major peaks located at BEs of 1021.8 and 1045.0 eV, which are attributed to the Zn 2p_3/2_ and Zn 2p_1/2,_ respectively, implying Zn^2+^ is in the composite [26]. In comparison to the reported values for pure ZnO [27], the Zn 2p of the composites shows a positive shift, which may be related to the electron transfer from the Fermi level of ZnO to that of Fe_2_O_3_ [28]. The O 1s core level spectrum is fitted into three distinct peaks. Specifically, the peaks at 530.3 and 531.8 eV correspond to the metal–oxygen bonds in both Fe_2_O_3_ and ZnO [29,30], while the peak at higher energy of 532.3 eV is attributed to the chemisorbed, dissociated oxygen or OH species on the surface of ZnO/Fe_2_O_3_ NFs [31].

**Figure 3:**
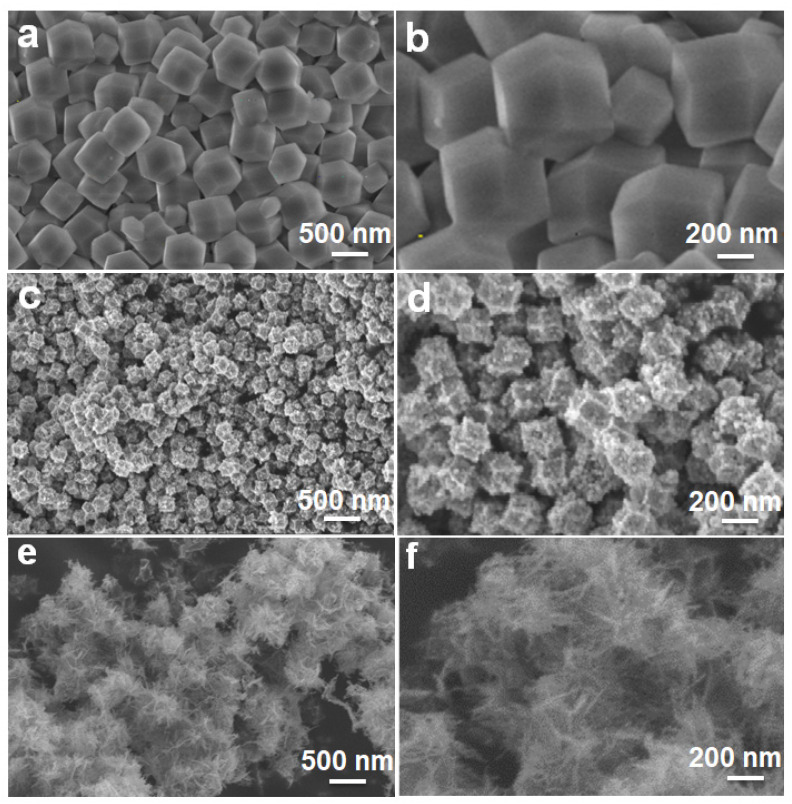
FESEM images of (**a**,**b**) ZIF-8, (**c**,**d**) ZnO and (**e**,**f**) ZnO/Fe_2_O_3_ NFs.

The morphological and structural information of the as-prepared samples were further characterized by the FESEM observation, as demonstrated in Figure 3. Typically, the ZIF-8 precursor displays a well-defined rhombic dodecahedral shape with sharp edges and smooth surfaces, as well as a relatively uniform size distribution of 500 nm on average, as seen in Figure 3a,b. After further thermally annealing the ZIF-8 at 420 °C, ZnO is obtained. Although it still retains the polyhedral shape of the ZIF-8 precursor, the size shrinks to 200 nm (Figure 3c). This is probably attributed to the pyrogenic decomposition. Moreover, compared to the smooth surface of the ZIF-8 precursor, the converted ZnO appears to possess a rough surface, and many tiny nanoparticles of ~20 nm in size can be clearly found on the surface (Figure 3d). Afterwards, the cautious dropwise addition of FeSO_4_ into the ZnO suspension at 0 °C inevitably results in the partial collapse of the polyhedral structures (Figure S1, ESI). With the subsequent annealing process at 500 °C, a more serious structure collapse occurs, and the resulted ZnO/Fe_2_O_3_ sample exhibits the fluffy flower-like architecture, as shown in Figure 3e,f.

**Figure 4:**
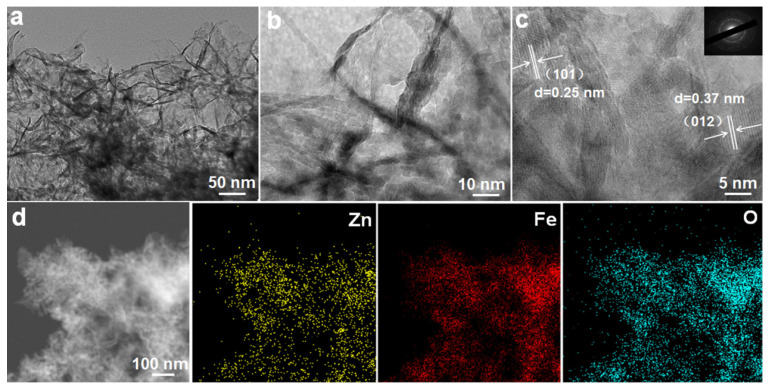
(**a**,**b**) TEM and (**c**) HRTEM images, SAED pattern (the inset in panel **c**), (**d**) STEM and corresponding elemental mapping images for the ZnO/Fe_2_O_3_ NFs.

The detailed structural features of the ZnO/Fe_2_O_3_ composite are further characterized by (HR)TEM. As exhibited in Figure 4a, the low-magnification TEM image evidences that ZnO/Fe_2_O_3_ NFs are constructed with numerous nanoflakes. The obvious wrinkles reveal the ultrathin nature of these flakes, and the average thickness is just several nanometers. Of particular note, numerous mesopores are clearly located in these nanoflakes (Figure 4b,c). The HRTEM image (Figure 4c) presents the well-defined lattice spacings of about 0.25 and 0.37 nm, which can be assigned to the (101) plane of the hexagonal ZnO and the (012) plane of the rhombohedral corundum Fe_2_O_3_. In addition, the corresponding SAED pattern (the inset in Figure 4c) indicates the polycrystalline nature of the nanoflakes. To reveal the spatial distribution of different elements in the ZnO/Fe_2_O_3_ NFs, elemental mapping analysis was conducted through the STEM mode. The homogeneous distributions of zinc, iron, and oxygen throughout the selected area are evident, as shown in Figure 4d. The EDX spectrum (Figure S2, ESI) further confirms the co-existence of Fe, Zn, and O elements in the composite, and specific contents of the three elements are summarized (Table S1, ESI). Thus, the relative weight content of the ZnO in the composite can be estimated as ~36.0 wt.%.

**Figure 5:**
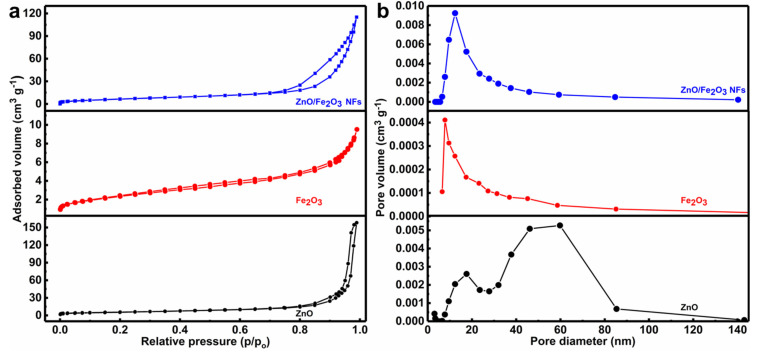
(**a**) N_2_ sorption isotherms and (**b**) pore size distribution plots of ZnO/Fe_2_O_3_ NFs, Fe_2_O_3_, and ZnO as indicated.

The porous characteristics of the as-prepared materials were further investigated by the N_2_ adsorption-desorption isotherms. As can be seen from Figure 5a, all the curves exhibit distinct isotherms with hysteresis loops, indicating their porous nature [32,33,34]. The BET technique was used to determine the SSA values of as-synthesized materials. Thanks to the porous structure, the as-prepared ZnO/Fe_2_O_3_ NFs are endowed with a high surface area of 35.5 m^2^ g^−1^, which is even larger than both ZnO (20.5 m^2^ g^−1^) and Fe_2_O_3_ (6.3 m^2^ g^−1^). Figure 5b profiles the corresponding pore size distribution plots of the three, which are calculated with the BJH method using the desorption branch of the nitrogen isotherms. Clearly, the ZnO/Fe_2_O_3_ sample illustrates a comparatively broad size distribution in the range of 5–37 nm, which renders a large pore volume (PV) (0.184 cm^3^ g^−1^) and average pore size (APS) of 2.9 nm. By contrast, the ZnO possesses a PV of (0.248 cm^3^ g^−1^) and APS of 4.8 nm, along with the PV of 0.012 cm^3^ g^−1^ and APS of 6.9 nm for the Fe_2_O_3_. It is the large SSA and PV that render the ZnO/Fe_2_O_3_ with a high adsorption capacity towards pollutants, favoring the enhanced photocatalytic activities [35,36].

### 3.2. Optical Studies

**Figure 6:**
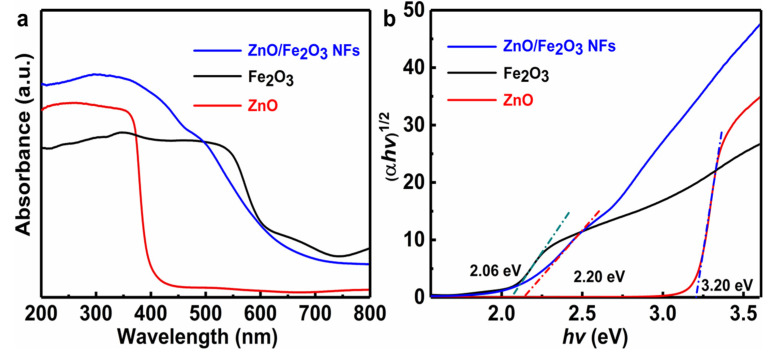
(**a**) UV-vis diffuse reflectance spectra and (**b**) Tauc plots of ZnO, Fe_2_O_3_, and ZnO/Fe_2_O_3_ NFs.

In general, the optical absorption ability of a semiconductor is closely relevant to its electronic structure and band gap and even affects the migration of electrons. Thus, it is commonly recognized as an indispensable factor to reflect its photocatalytic activities [8,36]. The optical absorption property of the ZnO/Fe_2_O_3_ NFs is investigated via UV-vis diffuse reflectance spectroscopy (DRS), and the corresponding result is displayed in Figure 6a. Typical spectra of pure ZnO and Fe_2_O_3_ are also plotted in Figure 6a for comparison. Remarkably, the phase-pure ZnO sample illustrates a fundamental absorption edge at 388 nm, which is consistent with the previous result [11]. As for the Fe_2_O_3_, the significant absorption in the visible light region is distinctive. Notably, the ZnO/Fe_2_O_3_ composite sample shows the absorption with an even stronger intensity in the visible light region than the ZnO, and the absorption edge presents an obvious red shift. The difference here probably results from the introduction of the Fe_2_O_3_ phase into ZnO. The red shift in the absorption spectrum of ZnO/Fe_2_O_3_ NFs indicates a strong interaction between ZnO and Fe_2_O_3_, accounting for the narrowing of the band gap [13]. Simultaneously, the electronic structure of the mixed phase may be altered greatly with respect to that of any single component, which is conducive to the enhanced photocatalytic performance [36,37,38,39]. According to the absorption spectra (Figure 6a), the plots of (αh*v*)^1/2^ versus photon energy (h*v*) of the as-prepared samples are profiled in Figure 6b. The band gap energy (*E_g_*) values can be estimated from the intercepts of the linear region in the plots of (αh*v*)^1/2^ on the *Y*-axis versus photon energy (h*v*) on the *X*-axis, as plotted in Figure 6b. Obviously, the *E_g_* value of the ZnO/Fe_2_O_3_ NFs is 2.20 eV, which is located between the ZnO (3.20 eV) and Fe_2_O_3_ (2.06 eV). This suggests that the suitable band gap of ZnO/Fe_2_O_3_ NFs can be potentially activated by visible light for photocatalytic degradation of organic contaminants.

### 3.3. Photochemical Performance Evaluation

**Figure 7:**
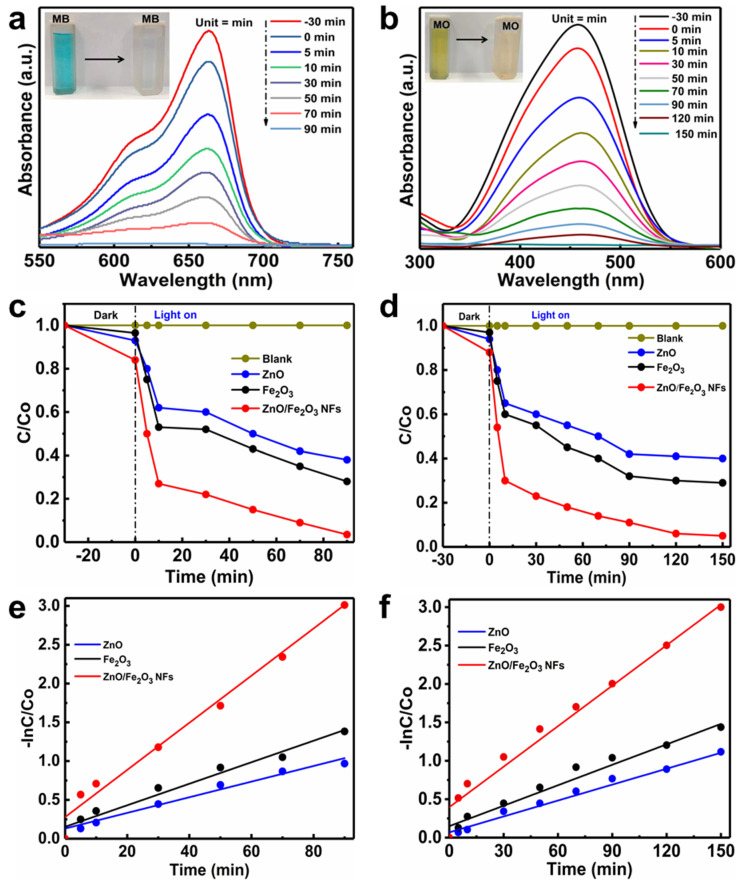
(**a**,**b**) Absorption spectra, (**c**,**d**) photodegradation curves, and (**e**,**f**) photodegradation reaction kinetic linear simulation curves under UV light irradiation for (**a**,**c**,**e**) MB and (**b**,**d**,**f**) MO with the photocatalysts of ZnO/Fe_2_O_3_ NFs, ZnO and Fe_2_O_3_.

The MB and MO are often used as models to evaluate the effectiveness of photocatalysts in the degradation of dye pollutants in wastewater [40,41,42]. The temporal evolution of absorption spectra of MB (Figure 7a) and MO (Figure 7b) over ZnO/Fe_2_O_3_ NFs is plotted as a function of the irradiation time. The characteristic absorption peaks of organic dyes (λ = 664 nm for MB and λ = 464 nm for MO) are applied to monitor the photocatalytic degradation process. Obviously, the absorption intensities of MB and MO both decrease gradually under UV light irradiation, which suggests the apparent degradation of the two dyes. As depicted in Figure 7a, the MB is almost decomposed over the ZnO/Fe_2_O_3_ NFs with the UV light irradiation for 90 min. While for the MO, the irradiation time of 150 min is needed for degradation (Figure 7b), revealing its weaker photocatalytic activities towards the MO when compared with MB. Moreover, the digital photographs (the insets in Figure 7a,b) visualize the color changes of MB and MO solutions from the deep color to the colorless, revealing that both MB and MO have been degraded. The above analysis clearly authenticates that the as-prepared ZnO/Fe_2_O_3_ composite holds enormous potential in wastewater treatment as an outstanding photocatalyst.

Furthermore, the photocatalytic degradation rates of MB and MO over ZnO/Fe_2_O_3_ NFs under UV light irradiation were also investigated. Figure 7c manifests the relative concentration (*C*/*C*_0_) of the MB as a function of time, where *C* is the concentration of MB at irradiation time *t* and *C*_0_ is its initial concentration. For comparison, the photocatalytic performance of ZnO and Fe_2_O_3_ under UV light irradiation is separately examined as well in the same system under identical experimental conditions. To elucidate the effects of adsorption, photolysis and photocatalysis, the control experiments of the adsorption decolorization with photocatalysts in the dark and photocatalytic degradation of MB under UV light irradiation in the presence or absence of the ZnO/Fe_2_O_3_ NFs are conducted in detail. Prior to UV light irradiation, the adsorption properties of the as-prepared ZnO/Fe_2_O_3_ NFs are initially studied. As plotted in Figure 7c, the adsorption-desorption equilibrium of photocatalysts with MB is established after 30 min in the dark. The ZnO/Fe_2_O_3_ NFs exhibit the strongest adsorption decoloration of ~16% when the equilibrium is reached. The stronger adsorption properties of ZnO/Fe_2_O_3_ NFs may be ascribed to their higher BET SSA and pore volume. Of especial note, the blank tests (just MB without any catalysts) under UV light reveal negligible photolysis, and only the limited MB can be degraded under UV light irradiation, suggesting the relative stability of MB upon UV light irradiation [43]. However, in the presence of a photocatalyst assisted by UV light illumination, the degradation efficiencies of MB over the photocatalysts are enhanced significantly, which signifies that the degradation of MB in the present study is indeed mainly through a photocatalytic process and/or photocatalysis. The experiments also highlight that the as-prepared ZnO/Fe_2_O_3_ NFs possess the strongest photocatalytic activities, where the photocatalytic degradation ratio of MB is up to as high as nearly 100% just after the UV light irradiation for 90 min, much better than those of ZnO (62%) and Fe_2_O_3_ (72%) with the same irradiation time.

Figure 7d summarizes the photodegradation performance of MO catalyzed by ZnO, Fe_2_O_3_, and ZnO/Fe_2_O_3_ NFs under UV light irradiation, along with the blank test. In the absence of the photocatalysts, the direct photolysis of MO was inappreciable under UV light irradiation, verifying the high stability of MO under UV light irradiation. During the photocatalytic experiments, an adsorption/desorption equilibrium is equally obtained between photocatalyst and MO before UV light irradiation. It is worthy of mentioning that the ZnO/Fe_2_O_3_ NFs also possess superior adsorption ability towards MO, compared to bare ZnO and Fe_2_O_3_, and the adsorption decoloration can reach 12% for the composite. With the concerted aid of ZnO and 150-min UV light irradiation, about 60% of MO is decomposed, less than Fe_2_O_3_ (71%) during the same period. More attractively, a maximum photocatalytic activity of ZnO/Fe_2_O_3_ NFs is found to be 95% for photocatalytic degradation of MO under the same conditions, confirming that the synergistic effect of Fe_2_O_3_ to ZnO effectively promotes the degradation of MO. The results above fully corroborate that ZnO/Fe_2_O_3_ NFs possess enhanced UV-light photocatalytic activity for the degradation of both MB and MO. Moreover, the photocatalytic efficiencies of our ZnO/Fe_2_O_3_ NFs observed here are even higher or competitive to other photocatalysts reported before (Table S2, ESI).

To further gain an in-depth understanding of reaction kinetics of the dye degradation catalyzed by the photocatalysts, the experimental data are fitted by a first-order model, as expressed by the equation: −ln(*C*/*C*_0_) = *Kt*, where *K* and *t* are the rate constants of the photocatalytic degradation, and irradiation time, respectively. The plot of ln(*C*/*C*_0_) versus *t* can be obtained by converting the photocatalytic degradation data, and the slope of the linear fitting curves is the *K* value. The converted kinetic plots of MB and MO are shown in Figure 7e,f, respectively, and corresponding *K* values of the two samples are summarized (Table S3, ESI). Indeed, the combination of Fe_2_O_3_ and ZnO has strong influences on the dye degradation rate. The ZnO/Fe_2_O_3_ NFs present about 3 and 2 times more reactive towards the degradation of MB than ZnO and Fe_2_O_3_, respectively, under UV light irradiation. Similar behaviors are also obtained over the decomposition of MO, and the ZnO/Fe_2_O_3_ composite material is approximately 4 and 2.6 times more efficient than pure ZnO and Fe_2_O_3_, respectively. The observed results here confirm the composite with the junction structure benefits for higher photocatalytic activities when compared to single-component ZnO and Fe_2_O_3_. It is in agreement with the contributions reported before, like ZnO-Fe_2_O_3_, g-Fe_2_O_3_/ZnO, and α-Fe_2_O_3_/ZnO systems [13,14,44].

**Figure 8:**
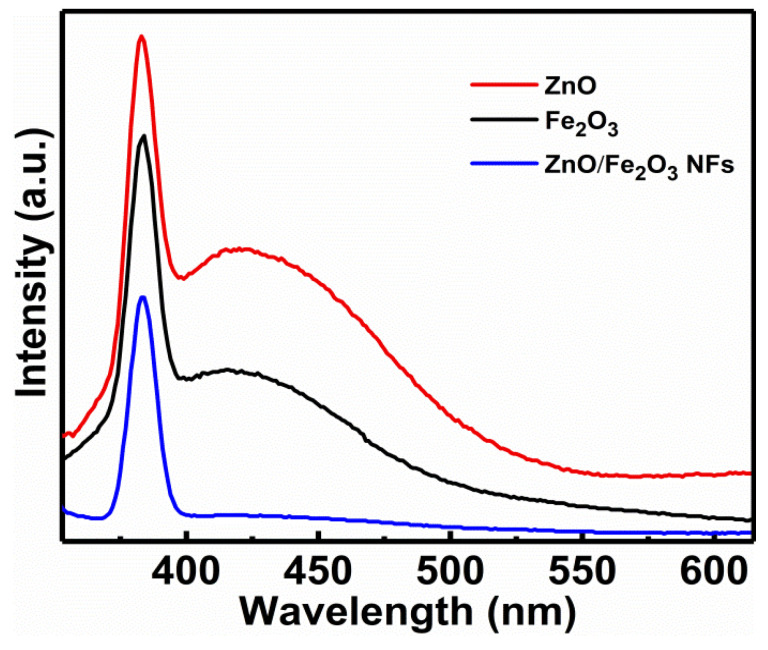
PL spectra of the as-prepared samples as indicated.

Although the BET surface area of a photocatalyst has been considered as an important factor in determining its photocatalytic activity, the separation efficiency of electron-hole pairs of the composite photocatalyst used in the research is always the key factor. Normally, the PL is recognized as an effective approach to obtain valuable information about the migration and recombination efficiency of the photo-generated electron-hole pairs, as the recombination of excited electrons and holes in a semiconductor is responsible for its PL emission [45,46,47]. The observation of stronger PL intensity indicates a greater prevalence of electron-hole recombination, while an increase in electron-hole recombination will decrease the concentrations of reductant/oxidant and dye degradation rate meanwhile. Figure 8 illustrates the RT PL emission spectra of the as-fabricated samples at an excitation wavelength of 300 nm. As is evident, the bare ZnO and Fe_2_O_3_ both show sharp UV emission peaks mainly located at 370 nm and broad visible emission peaks with the wavelength range from 400 to 550 nm, while the ZnO/Fe_2_O_3_ NFs just demonstrate a sharp UV emission peak centered at ~390 nm. The sharp UV emission peaks are normally associated with the band-edge emission resulting from the recombination of free excitons [48,49]. The generation of wide emission peaks in the visible-light area is generally attributed to the defects of metal atoms and oxygen vacancies during the crystal growing process [50,51]. The PL intensity of ZnO/Fe_2_O_3_ NFs is even lower than that of pure ZnO and Fe_2_O_3_, which definitely supports one fact that the recombination of photo-generated charge carriers is significantly inhibited for the case of ZnO/Fe_2_O_3_ NFs.

### 3.4. Photocatalytic Reaction Mechanism

**Figure 9:**
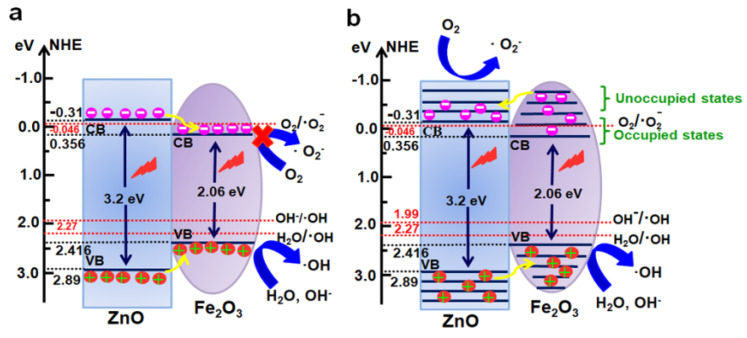
Schematic illustration for band-gap structure and transport of charge carriers of the ZnO/Fe_2_O_3_ NFs during the UV light irradiation: (**a**) isotropic double charge transfer mechanism and (**b**) reverse double charge transfer mechanism.

In general, photocatalytic activities of any catalyst are closely related to the redox abilities of electrons and holes [52,53], while its band potentials play important roles in its photocatalytic performance. The conduction band minimum energy (*E_CBM_)* and valence band maximum energy (*E_VBM_*) of the ZnO/Fe_2_O_3_ composite can be estimated by the following equations [1].

(1) $$E_{CBM}=X-E_{e}-0.5E_{g}$$

(2) $$E_{VBM}=E_{g}+E_{CB}$$

where *E_CB_* and *E_VB_* are the conduction and valence band edge potentials, respectively, *E_e_* stands for the energy of a free electron versus the normal hydrogen electrode (4.5 eV vs. NHE), *E_g_* denotes the band gap of a semiconductor, and *X* represents the absolute electronegativity of a semiconductor and can be expressed as the geometric mean of the absolute electronegativity of the constituent atoms. The *X* values for ZnO and Fe_2_O_3_ are 5.79 and 5.88 eV, respectively. Thus, *E_VB_* values of ZnO and Fe_2_O_3_ are calculated to be 2.89 and 2.416 eV (*vs.* NHE), respectively, and their corresponding *E_CB_* values are −0.31 and 0.356 eV (*vs.* NHE), respectively. As consistent with the literature [54], the conduction and valence band edges of Fe_2_O_3_ lie between those of ZnO in the composite (Figure 9a). Due to the narrower band gap and strong light absorption ability in the UV light regions of ZnO and Fe_2_O_3_ semiconductors, the photoelectrons can jump from the respective VB to CB of ZnO and Fe_2_O_3_ under the UV light irradiation, while the holes stay in their respective VB. As shown in the traditional charge migration mechanism (Figure 9a), the photogenerated electrons and holes both transfer from ZnO to Fe_2_O_3_. Thus, all electrons and holes would only stay within the sole Fe_2_O_3_ component of the composite, and their recombination probability is greatly increased. In particular, the electrons accumulated on the CB of Fe_2_O_3_ cannot reduce the O_2_ molecules adsorbed on the catalyst surface to produce •O_2_^−^ because of its more positive *E*_CB_ potential than *E*(O_2_/•O_2_^−^) (−0.046 eV vs. NHE) [55], and only the slightly left holes on the VB of Fe_2_O_3_ can generate reactive species of •OH due to its more positive *E_VB_* than *E*(H_2_O/•OH, 2.27 eV vs. NHE) and *E*(OH^−^/•OH, 1.99 eV vs. NHE) [56]. Based on the above analysis, the introduction of Fe_2_O_3_ into ZnO may result in the decrease in concentration of reactive species, and photocatalytic activities are weakened. However, it is obviously contrary to the aforementioned photocatalytic results, as displayed in Figure 7.

In the photocatalytic degradation process, a series of reactive oxygen species, such as h^+^, •OH, or •O_2_^−^, are normally supposed to be involved in the photocatalytic process [57,58]. In order to investigate why the ZnO/Fe_2_O_3_ composite demonstrates more efficient than pure ZnO towards the degradation of organic dyes, the reactive species trapping and hydroxyl radical quantification experiments were conducted. The ethylene diamine tetraacetic acid (EDTA-2Na), isopropanol (IPA), and benzoquinone (BQ) acted as the scavengers for h^+^, •OH, and •O_2_^−^ are introduced into the photocatalytic degradation process, respectively. The photocatalytic activities of ZnO/Fe_2_O_3_ NFs are investigated on the degradation of MB and MO with different scavengers (Figure S3, ESI). Notably, the photocatalytic degradation of both MB and MO is remarkably inhibited with the addition of EDTA-2Na, IPA and BQ. The results suggest that the h^+^, •OH, and •O_2_^−^ are the main active species in the photocatalytic process under UV light irradiation.

As a result, the traditional charge transfer mechanism cannot explain the enhanced photocatalytic performance of ZnO/Fe_2_O_3_ composite at all, as well as the existing main active species in the photocatalytic process here. Thus, a plausible reverse charge transfer mechanism in ZnO/Fe_2_O_3_ NFs is tentatively proposed, as schematically described in Figure 9b. In the charge transfer mechanism, the strong electronic interaction between ZnO and Fe_2_O_3_ is not limited to the occupied states but the unoccupied levels. As presented in Figure 9b, the unoccupied ones of Fe_2_O_3_ demonstrate more negative potential than the *E_CB_* potential of ZnO. Under the UV light irradiation, the electrons are exited from the respective VB to CB of ZnO and Fe_2_O_3_. As for the Fe_2_O_3_ semiconductor, the excited electrons not only exist in the occupied states but in the unoccupied levels. The band potential of the unoccupied levels for Fe_2_O_3_ is lower than the *E_CB_* potential of ZnO. In the reported literature [12], arising from the formation of the heterojunction between ZnO and Fe_2_O_3_ semiconductors, the photogenerated electrons in Fe_2_O_3_ can transfer to ZnO until the Fermi levels of ZnO and Fe_2_O_3_ equalize. Thus, they can result in the accumulation of negative charges in CB of ZnO. At the same time, the holes transfer from the VB of ZnO to that of Fe_2_O_3_. Moreover, the CB potential of ZnO (−0.31) is less positive than the oxidative standard potential of O_2_/•O_2_^−^ (−0.046 eV vs. NHE), suggesting that the collected electrons in the CB of ZnO can reduce O_2_ to produce active species of •O_2_^−^. Accordingly, the VB potential of Fe_2_O_3_ (2.416 eV) is more positive than the reductive standard potentials of H_2_O/•OH (2.27 eV vs. NHE) and OH^−^/•OH (1.99 eV vs. NHE) [59], which indicates that the holes in the VB of Fe_2_O_3_ can oxidize the H_2_O or OH^−^ into the reactive specie of •OH. Meanwhile, the VB hole of Fe_2_O_3_ can directly oxidize MB and MO due to the much higher redox potential of *E_VB_* (Fe_2_O_3_) than MB (1.08 eV) and MO (1.48 eV). In brief, the high interface quality at the junction will contribute to the mobility of the photogenerated electrons and holes, increasing the photocatalytic activities of ZnO/Fe_2_O_3_ NFs.

**Figure 10:**
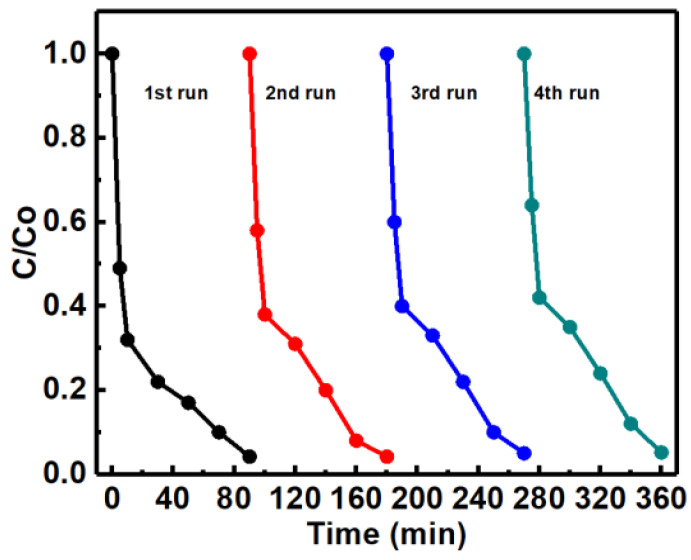
Cycling stability of ZnO/Fe_2_O_3_ NFs for degradation of MB.

**Figure 11:**
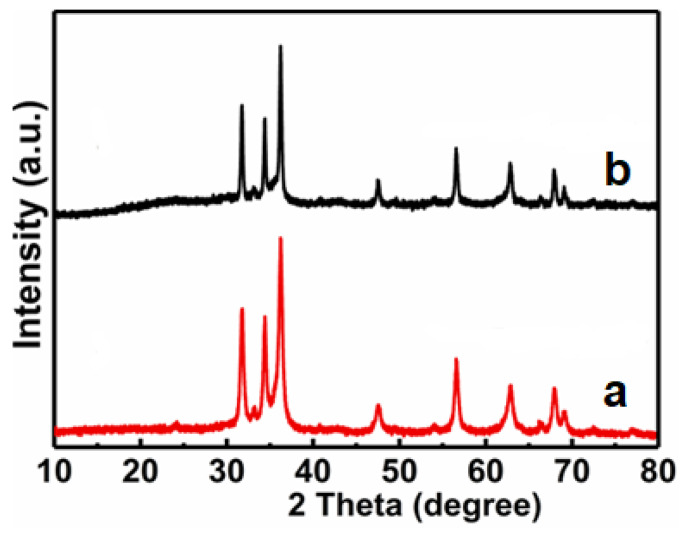
XRD patterns of ZnO/Fe_2_O_3_ NFs (**a**) before and (**b**) after four cycles for photocatalytic degradation of MB.

The excellent reusability of any photocatalyst is also recognized as a key factor for its practical applications. To evaluate the photocatalytic stability of ZnO/Fe_2_O_3_ NFs, the photocatalytic activities of ZnO/Fe_2_O_3_ NFs are investigated by circulating runs in the degradation of the MB under UV light irradiation. As can be observed from Figure 10, the shrinking in degradation rate is still kept within ~1% even up to four consecutive cycles under the same photocatalytic conditions. The decrease in the degradation efficiency is probably attributed to the loss of photocatalysts during the cycling process. Additionally, the XRD pattern of ZnO/Fe_2_O_3_ NFs after the four-cycle photocatalytic degradation of the MB presents no obvious change in comparison with its original pattern (Figure 11), which further verifies the remarkable stability of the resultant ZnO/Fe_2_O_3_ NFs. The observation here implies that ZnO/Fe_2_O_3_ NFs own superior cycling stability, and no obvious photocorrosion takes place during the photocatalytic degradation of the MB molecules, which is of particular significance for its commercial applications.

## 4. Conclusions

In conclusion, a facile and efficient method was developed to synthesize ZnO/Fe_2_O_3_ NFs by using the ZIF-8 as a precursor. Compared to the phase-pure ZnO and Fe_2_O_3_, the obtained ZnO/Fe_2_O_3_ NFs showed even higher photocatalytic activities in the photocatalytic degradation of both MB and MO under UV light irradiation due to its higher SSA/PV, stronger light absorption property, lower recombination rate of photogenerated electrons and holes, and better-quality interface between ZnO and Fe_2_O_3_. Meanwhile, the h^+^, •OH, and •O_2_^−^ were the main reactive species responsible for the photocatalytic degradation of MB and MO. The in-depth understanding of the photocatalytic degradation mechanism for the enhanced photocatalytic activities of the ZnO/Fe_2_O_3_ NFs was also reasonably put forward. Additionally, the resultant ZnO/Fe_2_O_3_ NFs exhibited remarkable stabilities for practical applications. It highlights that our ZnO/Fe_2_O_3_ NFs are promising photocatalysts for photocatalytic degradation of organic pollutants and beyond.

## Supplementary Materials

The following are available online at https://www.mdpi.com/article/10.3390/nano11123239/s1, Figure S1: SEM of the sample before annealing for synthesizing ZnO /Fe_2_O_3_ NFs, Figure S2: EDX spectrum of ZnO/Fe_2_O_3_ NFs, Figure S3: Active species trapping experiments using the ZnO/Fe_2_O_3_ NFs as the photocatalyst under UV light irradiation: (a) MB (1 mg L^−1^) and (b) MO (1 mg L^−1^), Table S1 The elemental composition of Zn, O, and Fe in the ZnO/ Fe_2_O_3_ NFs, Table S2 Comparison in degradation efficiency of MB and MO dyes using the ZnO/Fe_2_O_3_ NFs as electrocatalyts with other catalysts, Table S3 Values of *k* for MB and MO dyes using different photocatalysts.
