# Supplementary material for: MOFs Derived Hetero-ZnO/Fe2O3 Nanoflowers with Enhanced Photocatalytic Performance towards Efficient Degradation of Organic Dyes
Source: Nanomaterials (Basel). 2021 Nov 29;11(12):3239. doi: 10.3390/nano11123239 (PMC8707436; doi:10.3390/nano11123239)
Supplement: Supplementary file 1 [file nanomaterials-11-03239-s001.zip › nanomaterials-1467652-supplementary.pdf]

Supporting Information

MOFs derived hetero-ZnO/Fe_2O_3 nanoflowers with enhanced photocatalytic performance towards efficient degradation of organic dyes

Fakhr uz Zaman, Bing Xie, Jinyang Zhang, Tianyu Gong, Kai Cui, Linrui Hou,* Jiali Xu, Zhirou Zhai, Changzhou Yuan*

School of Materials Science & Engineering, University of Jinan, Jinan 250022, P. R. China

*Corresponding authors: mse_houlr@ujn.edu.cn; houlr629@163.com (*Prof.* Hou). mse_yuancz@ujn.edu.cn (*Prof.* Yuan).

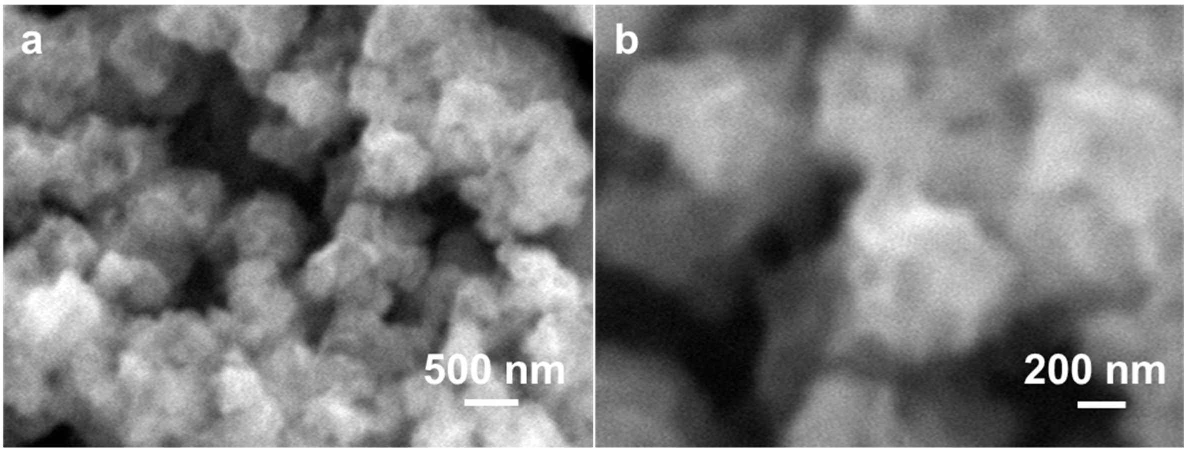

Figure S1. SEM of the sample before annealing for synthesizing ZnO /Fe_2O_3 NFs.

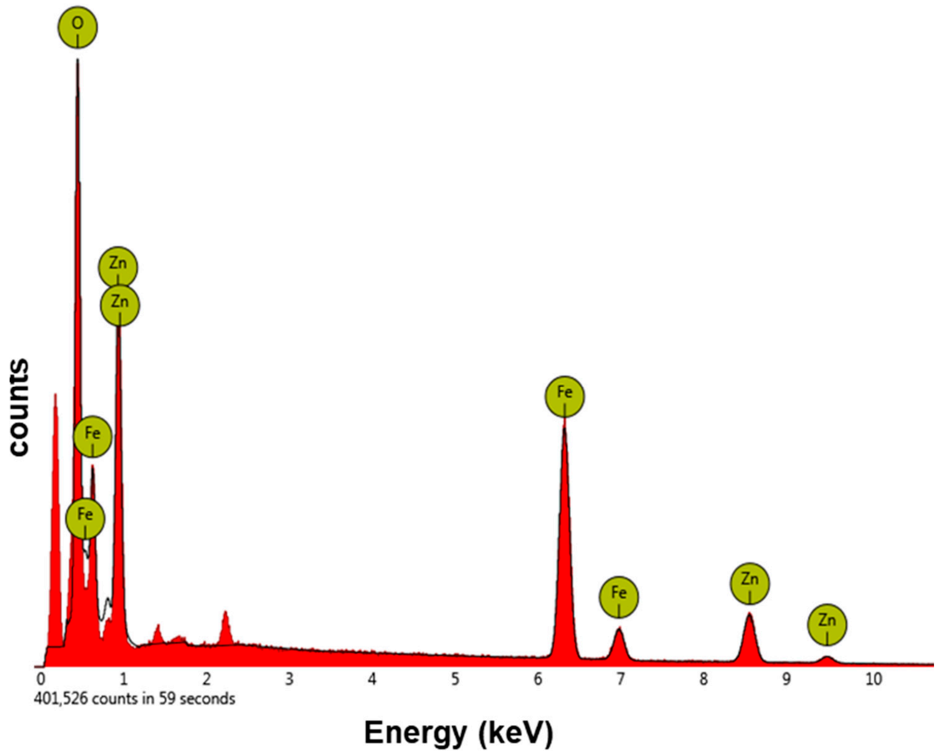

Figure S2. EDX spectrum of ZnO/Fe_2O_3 NFs.

Table S1 The elemental composition of Zn, O, and Fe in the ZnO/ Fe_2O_3 NFs.

Element Symbol	Element Name	Weight Conc.
O	Oxygen	30.88
Fe	Iron	42.01
Zn	Zinc	27.11

Table S2. Comparison in degradation efficiency of MB and MO dyes using the ZnO/Fe_2O_3 NFs as electrocatalyts with other catalysts.

composite	Light	Dyes		Irradiation time(min)	Percent Degradation	Ref
		MB	MO			
ZnO/Fe_2O_3	UV	/	MO	140 min	70%	1
ZnO/Fe_2O_3	Visible	MB	MO	50 and 80 min	95 and 82.5%	2
ZnO-modified g-C_3N_4	200W	MB	/	120 min	90%	3
ZnO/MOS_2	UV-vis	MB	/	80 min	81%	4
Cu-ZnO/S-gC_3N_4	UV	MB	/	90 min	93%	5
Ag/ZnO	UV	MB	/	60 min	60%	6
ZnO/Cu_2O	UV	/	MO	180 min	73%	7
ZnO/SnO_2	UV	/	MO	100 min	56%	8
ZnO/Eu	Sun	/	MO	150 min	62%	9
Graphene/ZnO	Sun	/	MO	360 min	96%	10
ZnO/Fe_2O_3 NFs	UV	MB	MO	90 and 150 min	100 and 96%	This work

Table S3 Values of *k* for MB and MO dyes using different photocatalysts

Dyes	Sample	ZnO	Fe_2O_3	ZnO/Fe_2O_3
MB	k $(min)^{-1}$	0.0109	0.015	0.034
MO	k $(min)^{-1}$	0.006	0.0095	0.024

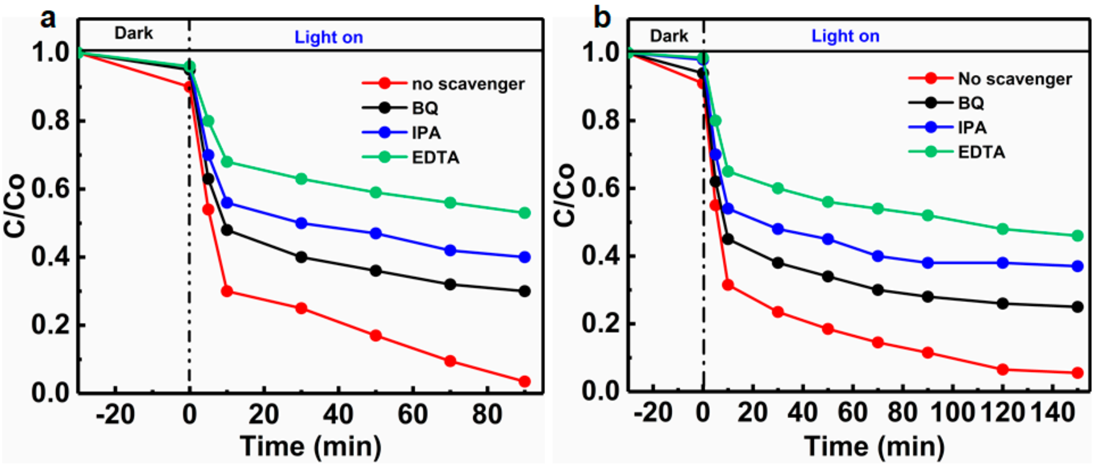

Figure S3. Active species trapping experiments using the ZnO/Fe_2O_3 NFs as the photocatalyst under UV light irradiation: (a) MB (1 mg L^{-1}) and (b) MO (1 mg L^{-1}).

References

1. Yan, W.; Fan, H.; Yang, C. Ultra-fast synthesis and enhanced photocatalytic properties of alpha Fe_2O_3/ZnO core-shell structure, Mater. Lett. 2011, 65, 11, 1595–1597
2. Liu, Y.; Yu, L.; Hu, Y.; Guo, C.; Zhang, F.; Lou, X. W. D. A magnetically separable photocatalyst based on nest-like γ-Fe_2O_3/ZnO double-shelled hollow structures with enhanced photocatalytic activity, *Nanoscale* **2012**, *4*, 1, 183-187.
3. Paul, DR.; Gautam, S.; Panchal, P.; Nehra, SP.; Choudhary, P.; Sharma, A. ZnO-Modified g-C_3N_4: A Potential Photocatalyst for Environmental Application. ACS Omega. 2020, 5, 8, 3828-3838.
4. Krishnan, U.; Kaur, M.; Kaur, G.; Singh, K.; Dogra, AR.; Kumar, M.; Kumar, A. MoS_2/ZnO nanocomposites for efficient photocatalytic degradation of industrial pollutants, Mater. Res. Bull. 2019, 111, 212-221.
5. Javed, M.; Qamar, MA.; Shahid, S.; Alsaab, H.; Asif, S. Highly efficient visible light active Cu ZnO/S-gC_3N_4 nanocomposites for efficient photocatalytic degradation of organic pollutants, RSC Adv., 2021, 11, 59, 37254–37267.
6. Height, M.J.; Pratsinis, S.E.; Mekasuwandumrong, O.; Praserthdam, P. Ag-ZnO catalysts for UV photodegradation of methylene blue, Appl. Catal. B, 2006, 63, 3-4, 305-312.
7. Xu, C.; Cao, L.; Su, G.; Liu, W.; Liu, H.; Yu, Y.; Qu, X. Preparation of ZnO/Cu_2O compound photocatalyst and application in treating organic dyes. J. Hazard. Mater. 2010, 176, 1-3, 807-813.

8. Ali, W.; Ullah, H.; Zada, A.; Alamgir, M.K.; Muhammad, W.; Ahmad, M.J.; Nadhman, A. Effect of calcination temperature on the photoactivities of ZnO/SnO_2 nanocomposites for the degradation of methyl orange. Mater. Chem. Phys. 2018, 213, 259-266.
9. Trandafilović, LV.; Jovanović, DJ.; Zhang, X.; Ptasińska, S.; Dramićanin, M. Enhanced photocatalytic degradation of methylene blue and methyl orange by ZnO: Eu nanoparticles. Appl. Catal. B, 2017, 203, 740-752.
10. Hao, C.; Yang, Y.; Shen, Y.; Feng, F.; Wang, X.; Zhao, Y.; Ge, C. Liquid phase-based ultrasonic-assisted synthesis of G–ZnO nanocomposites and its sunlight photocatalytic activity. Mater. Des. 2016, 89, 864-871.
